# Molten-State Dielectrophoretic Alignment of EVA/BaTiO_3_ Thermoplastic Composites: Enhancement of Piezo-Smart Sensor for Medical Application

**DOI:** 10.3390/ijms232415745

**Published:** 2022-12-12

**Authors:** Omar Zahhaf, Giulia D’Ambrogio, Angela Giunta, Minh-Quyen Le, Guilhem Rival, Pierre-Jean Cottinet, Jean-Fabien Capsal

**Affiliations:** 1 Laboratoire de Génie Electrique et Ferroélectricité, Campus Ladoua, Institut National des Sciences Appliquées, Université de Lyon, 69621 Villeurbanne, France; 2 Department Materials Engineering and Nanotechnology, Politecnico di Milano, Campus Leonardo, 20133 Milan, Italy

**Keywords:** molten-state dielectrophoresis, thermoplastic composites, piezoelectric sensor performance, structured materials

## Abstract

Dielectrophoresis has recently been used for developing high performance elastomer-based structured piezoelectric composites. However, no study has yet focused on the development of aligned thermoplastic-based piezocomposites. In this work, highly anisotropic thermoplastic composites, with high piezoelectric sensitivity, are created. Molten-state dielectrophoresis is introduced as an effective manufacturing pathway for the obtaining of an aligned filler structure within a thermoplastic matrix. For this study, Poly(Ethylene-co Vinyl Acetate) (EVA), revealed as a biocompatible polymeric matrix, was combined with barium titanate (BaTiO_3_) filler, well-known as a lead-free piezoelectric material. The phase inversion method was used to obtain an optimal dispersion of the BaTiO_3_ within the EVA thermoplastic matrix. The effect of the processing parameters, such as the poling electric field and the filler content, were analyzed via dielectric spectroscopy, piezoelectric characterization, and scanning electron microscopy (SEM). The thermal behavior of the matrix was investigated by thermogravimetric analysis (TGA) and differential scanning calorimetry analysis (DSC). Thermoplastic-based structured composites have numerous appealing advantages, such as recyclability, enhanced piezoelectric activity, encapsulation properties, low manufacturing time, and being light weight, which make the developed composites of great novelty, paving the way for new applications in the medical field, such as integrated sensors adaptable to 3D printing technology.

## 1. Introduction

Thermoplastic polymers are highly versatile materials with a wide range of applications. They are extremely attractive because of their light weight, high mechanical strength and low processing cost [1,2,3]. Their low melting point allows them to be easily manufactured and molded on a large scale, as well as being easily recyclable [4,5]. They are suitable for a variety of manufacturing processes, including emerging 3D printing [6,7,8].

Electroactive properties, such as piezoelectricity, can be conferred to these thermoplastic polymers by adding the appropriate fillers, thus, leading to an electroactive composite [9,10]. Piezoelectric ceramic fillers, such as BaTiO_3_, have received considerable attention in both research and industrial fields, due to their low cost, biocompatibility and high piezoelectric sensitivity. When coupled with a suitable thermoplastic matrix, such as EVA or PLA, the filler/matrix combination leads to an extremely versatile and easily manufactured composite. Hence, piezoelectric thermoplastic composites are emerging as a novel and promising approach to more efficient structural monitoring sensors [11]. The coupled piezoelectric and structural properties make such composites interesting for a variety of applications, ranging through biomedical, aeronautical, and industrial systems [12,13,14,15,16,17]. Indeed, their wide-ranging potential lies in their diverse properties, such as flexibility, ease of processing, light weight and low cost. Thus, for sensor development, thermoplastic piezoelectric composites are an excellent compromise between piezoelectric and structural properties [18,19].

Nonetheless, it has been pointed out that composites with randomly dispersed fillers do not usually exhibit high enough sensitivities to compete with bulk ceramics [20,21,22] or expensive fluoropolymers (PVDF, PVDF-TrFE) [23,24,25]. These composites, often referred to as 0–3 composites, according to Newnham’s connectivity nomenclature [26], require high filler contents to generate sufficient output signal, thus, sacrificing their flexibility and low density. Therefore, in order to enhance the piezoelectric activity of such composites, structural improvement is necessary. A composite’s connectivity is undoubtedly a major factor influencing its properties [26]. Filler alignment within the polymer matrix has proven to be an efficient way to enhance a composite’s piezoelectric performance. Referred to as 1–3 composites, they incorporate fillers aligned in columnar structures along a preferential direction. The spatial arrangement of the ceramic particles influences the electric field distribution during the poling process. In the 1–3 connectivity, the ceramic fillers experience a higher electric field than those in the 0–3 composites, as the former are less shielded by the polymer matrix. In addition, the 1–3 connectivity results in a higher filler contribution along the direction of the applied electric field. Consequently, 1–3 structured piezocomposites are anisotropic, showing enhanced piezoelectric and dielectric properties along the alignment direction. 

Such connectivity can be achieved through a variety of methods, one of which is electric field-induced filler alignment. This process, known as dielectrophoresis, uses induced dipole interactions between dielectric fillers to align a random 0–3 dispersion into a 1–3 chain-like structure [27,28]. It results in a field-structured anisotropic composite, giving rise to enhanced piezoelectric properties in the field direction. Moreover, as the filler structuring within a composite induces its anisotropy, it is expected that the mechanical properties are affected as well. Indeed, as the fillers are aligned along the preferred direction (i.e., the z axis), the obtained columnar structure acts as reinforcement against compression stress. This enhances the elastic compression modulus along the alignment axis [29,30]. It is also noteworthy that dielectrophoresis can be implemented to structure various non-piezoelectric composites, such as thermally or electrically conductive materials, and even semiconductors [31,32,33]. Other techniques of structuration, like magnetophoresis or the light-induced plasticity process, also lead to a creation of anisotropic properties, which, in turn, substantially enhance the intrinsic properties (magnetic, mechanical, shape memory) of composites along a preferred direction [34,35,36,37,38]. 

Previous research studies, primarily focused on thermoset matrices, like polydimethylsiloxane (PDMS), polyurethane (PU) or epoxy, reported a significant improvement in piezoelectric properties achieved by dielectrophoresis [39,40,41]. Structuration of thermoplastic composites [42,43,44], however, has rarely been investigated in the literature. Although thermoset matrices usually exhibit suitable viscosities for dielectrophoretic structuring, the need for an additional curing stage is often time-consuming. In addition, only certain thermoset matrices can be used, as some of them have too short a curing time to ensure sufficient particle alignment. Accordingly, the thermosetting nature of the cross-linked structures in both PDMS and PU elastomers severely limits the commercial viability of their manufacture. Therefore, the use of a thermoplastic matrix paves the way for the development of a new generation of materials that are cost-effective, with manufacturing time savings, ease of production, high impact resistance, recyclability and adaptability to 3D printing technology [45,46,47]. Molten-state dielectrophoresis is, thus, introduced as an effective technique for structuring thermoplastic composites at high temperature, with the aim of enhancing their piezoelectric properties without impairing their flexibility, nor their manufacturing time and cost.

The purpose here was to study the enhancing effect of filler alignment on the piezoelectric and dielectric properties of thermoplastic composites. This research also aimed to investigate the influence of relevant parameters, such as the filler content and the poling electric field, on the composites’ properties. Finally, the thermal stability of the developed composites was assessed, based on a comparison with the existing piezoelectric materials. Thus, in this work, we introduce the Poly(Ethylene-co vinyl acetate) (EVA), a semi-crystalline thermoplastic polymer with a low enough melting point. In addition, EVA has been revealed to be biocompatible [48,49], making it one of the most excellent candidates for the development of piezoelectric sensors in the medical field. High performing structured composites (1–3 connectivity) were developed and compared to their standard unstructured counterparts (0–3 connectivity). Different volume fractions were investigated to assess enhancement of the connectivity between the filler and matrix phases. Differential Scanning Calorimetry was performed to analyze the matrix melting temperature, thus, identifying the optimal structuring and poling temperature. Thermogravimetric analysis and scanning electron microscopy were carried out to further characterize the matrix and the manufactured composites. Dielectric and piezoelectric measurements were performed on both structure types over different volume fractions, in order to highlight the superiority of the aligned composites. The results indicated a significant improvement in the piezoelectric charge coefficient along the alignment direction. Thanks to its surprising piezoelectric and physical properties, the designed material is of major novelty. The ability to shape it easily into complex structures makes aligned thermoplastic composites a stepping-stone to many new applications suitable for 3D printing technology. 

## 2. Results and Discussion

### 2.1. Thermogravimetric Analysis (TGA)

The TGA results provided a useful insight into EVA’s thermal degradation and stability. The thermal decomposition of pure EVA can be separated into two main stages. The first one, commonly referred to as “Deacetylation”, occurs in the temperature range of 300 to 400 °C and relates to the elimination of the vinyl acetate component of the copolymer. This step leads to the formation of acetic acid molecules and an ethylene structure on the rest of the carbon chain [50,51]. The second one, occurring above 400 °C, corresponds to the degradation of the polyethylene main chain. Since the first stage decomposition is tied to the presence of VA in the EVA copolymer, the deacetylation phase is more pronounced with an increasing VA content. Thus, it was possible to estimate, based on the weight loss of the first stage, the initial VA content present in the copolymer using the following equation [52]:

(1) $$VA\left(wt\%\right)=\frac{W_{VA}}{W_{AC}}\cdot W_{fd.loss\%}$$

where, VA(wt%) is the estimated VA content, $W_{VA}$ and $W_{AC}$ are respectively the molecular mass of VA and acetic acid, and $W_{fd.loss\%}$ is the weight loss of the first stage decomposition. 

**Figure 1:**
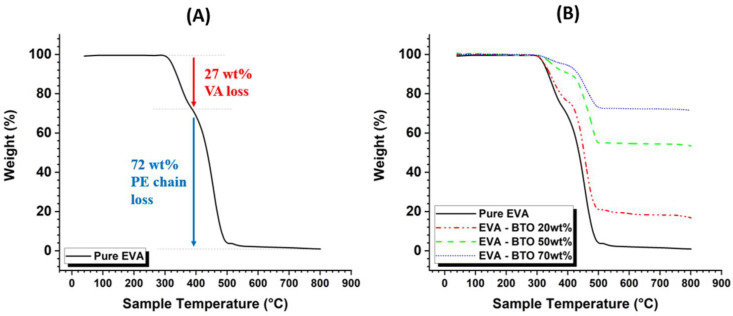
TGA curves of: (**A**) pure EVA, (**B**) EVA/BaTiO_3_ composites at different filler contents.

Figure 1A shows the thermal decomposition of pure EVA over the temperature range of 25 °C to 800 °C. Until nearly 300 °C, the pure EVA exhibited a good thermal stability as no weight loss was recorded. This result was relevant as it allowed confirmation that the structuring temperature (i.e., 130 °C) used throughout dielectrophoresis was far below the level of the two thermal decompositions described above. The first stage decomposition, corresponding to the elimination of the VA component, occurred between 300 °C and 400 °C, where the sample experienced around 27% weight loss. Substituting this value into Equation (1) yielded the initial VA content, i.e., equal to 39 wt%. The second decomposition phase occurred between 400 °C and 800 °C, where the weight drastically dropped; particularly beyond 500 °C, as the sample loss was almost the totality of its initial mass (~99%).

EVA/BaTiO_3_ unstructured composites were also analyzed by TGA, under a similar temperature range, as illustrated in Figure 1B. For all filler contents, the polymeric part of the composite followed the same decomposition path described above: a first decomposition stage of the VA component between 300 °C and 400 °C, followed by the second decomposition phase of the remaining PE chains between 400 °C and 500 °C. As expected, the higher the filler content of the composite, the lower the total weight loss. Since BaTiO_3_ is a ceramic material that does not undergo any thermal decomposition at 800 °C, its mass was assumed to be unchanged during the experiment. Therefore, based on the weight loss resulting from the complete decomposition of EVA (at 800 °C), it was possible to accurately determine the mass fraction of the ceramic fillers initially present in the composite. The results, displayed in Figure 1B, revealed three different final weight losses (in steady state) for different filler contents: 18 wt%, 54 wt% and 72 wt%, corresponding to 3 vol%, 16 vol% and 29 vol%, respectively. It is also interesting to note that the EVA/BaTiO_3_ composites, to some extent, exhibited similar two-phase decomposition at approximately the same temperature range.

### 2.2. Differential Scanning Calorimetry (DSC)

**Figure 2:**
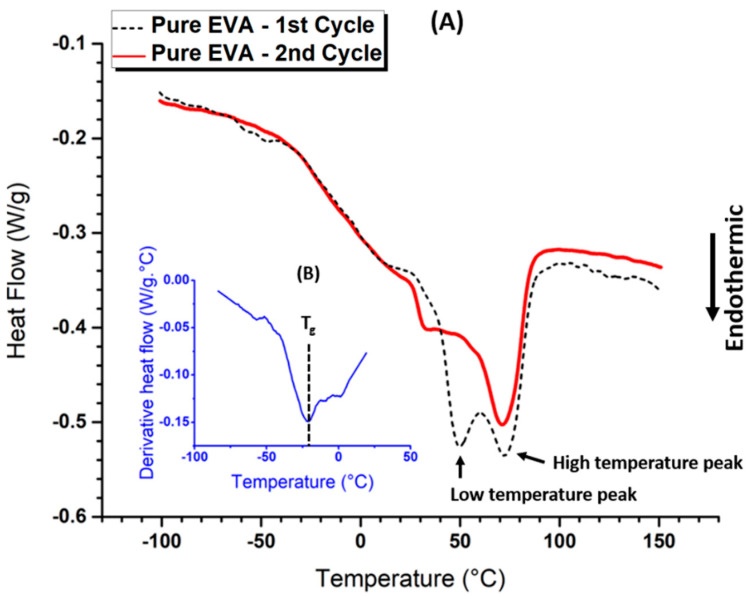
(**A**) DSC analysis of pure EVA-2 thermal cycles detailed in Figure 16, (**B**) the normalized heat flow derivative as a function of temperature.

Pure EVA, as well as EVA/BaTiO_3_ unstructured composites, were analyzed by DSC. As described above, two thermal cycle runs were carried out on each sample in order to disregard any prior thermal history. Figure 2A shows the results of the 1st and 2nd runs for the pure EVA. The first observed thermal event was the glass transition occurring throughout both cycles. It was represented by a broad endothermic step transition between −50 °C and 20 °C, approximately. This change in the heat capacity could be related to an increase in polymer chain mobility. From a mechanical point of view, this phenomenon was characterized by a transition from a rigid to a more flexible state. The glass transition temperature could then be estimated through a first order derivative of the normalized heat flow, shown in Figure 2B (blue line). The resulting derivative function described a downward peak where the minimum corresponded to the glass transition temperature. For pure EVA, the T_g_ was estimated to be −21.5 °C, approximately. The second recorded thermal event was crystal melting. Two endothermic peaks were observed in the 1st run (black dashed line in Figure 2A), a low temperature peak at 49 °C and a higher temperature peak at 72 °C. The presence of dual endotherm behavior was a consequence of bimodal distribution of crystallite size [53]. On the second run (red solid line in Figure 2), the endothermic double peak evolved into a shoulder at 50 °C, followed by a prominent peak at 71 °C. This change indicated the formation of smaller size crystallites as a result of the first thermal run. The integrated melting enthalpy (denoted $\Delta H_{mp}$) for the pure EVA was calculated to be 25 J/g, leading to an estimation of its degree of crystallinity ($\chi_{c})\;$ using the following expression [54]:

(2) $$\chi_{c}\left(\%\right)=\frac{\Delta H_{mp}}{\Delta H_{100}}\times 100$$

where $\Delta H_{100}$ is the specific melting enthalpy of 100% crystalline polyethylene (288 J/g) [55]. Thus, the degree of crystallinity of the pure EVA was 8.68%. 

**Figure 3:**
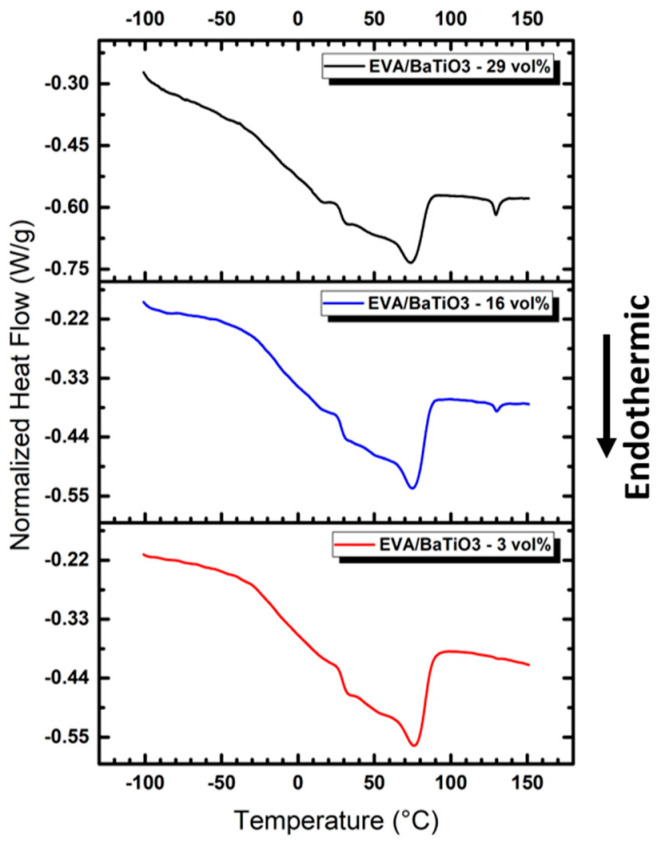
DSC analysis of EVA-BaTiO_3_ unstructured composites: 3 vol%, 16 vol% and 29 vol%.

Figure 3 presents the DSC curves of EVA/BaTiO_3_ unstructured composites elaborated with several particle ratios (i.e., 3 vol%, 16 vol%, 29 vol%). The results showed a similar behavior to the one of the pure EVA, observed in Figure 2. All three composites exhibited a glass transition occurring between −50 °C and 20 °C, and a melting transition characterized by a shoulder at 50 °C, followed by a peak at 75 °C. This similarity in the thermal behavior between the pure EVA and EVA/BaTiO_3_ unstructured composites suggested that the presence of BaTiO_3_ filler did not significantly alter the matrix’s physical structure. The melting transition was followed by an endothermic peak at 130 °C, barely visible in the 3 vol% and became more pronounced as the filler content increased. This thermal event corresponded to BaTiO_3_ phase transition from a ferroelectric tetragonal structure to a paraelectric cubic structure [56]. Table 1 summarizes the glass transitions and melting temperatures, as well as the integrated melting enthalpy normalized to the polymer mass as a function of particle volume fraction. As depicted in Table 1, when normalized to polymer mass, all unstructured composites exhibited similar melting enthalpies to the pure EVA, which allowed confirmation of the accuracy of the mass fractions determined in the previous section. Moreover, the composites showed slightly higher melting and glass transition temperatures, suggesting that the presence of BaTiO_3_ ceramic fillers did not significantly alter the polymer’s thermal properties. 

### 2.3. Scanning Electron Microscopy (SEM)

**Figure 4:**
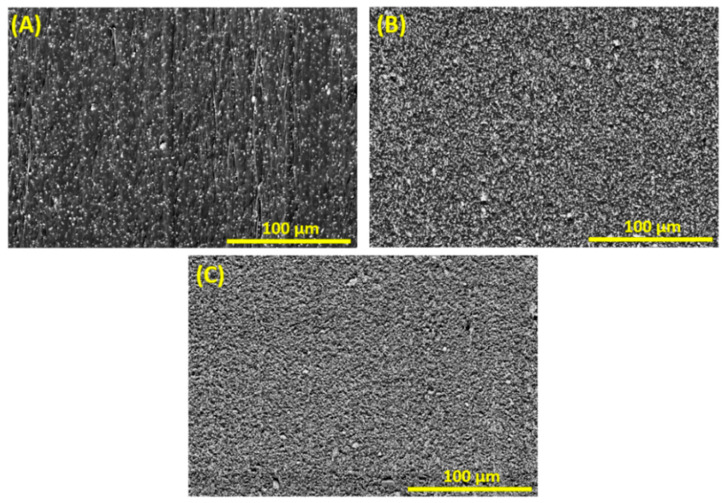
The 0–3 unstructured EVA/BaTiO_3_ composites with different volume fractions: (**A**) 3 vol%, (**B**) 16 vol%, (**C**) 29 vol%.

**Figure 5:**
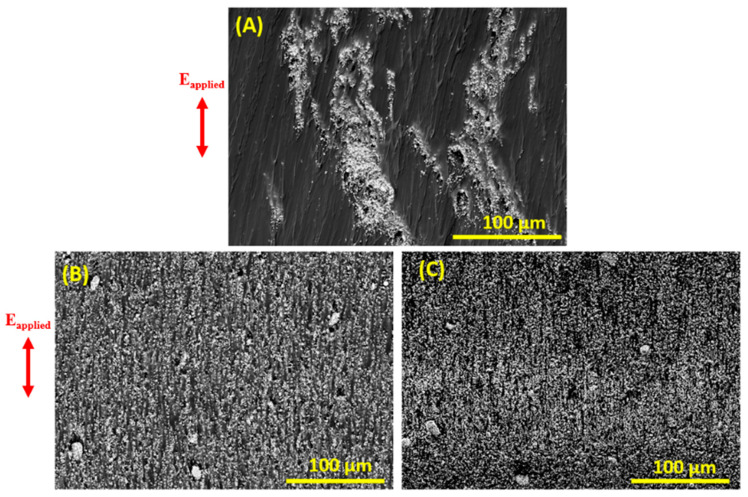
SEM micrographs of EVA/BaTiO_3_ composites structured at 6 kV·mm^−1^: (**A**) 3 vol% filler content, (**B**) 16 vol% filler content, (**C**) 29 vol% filler content.

Figure 4A–C show cross-sectional SEM observations of unstructured EVA/BaTiO_3_ composites where the BaTiO_3_ nanoparticles were homogeneously distributed in the EVA matrix with different volume fractions. As the filler content increased, it was clearly visible from the micrographs that the inter-particle distance decreased. Figure 5A–C illustrate cross-sectional observations of EVA/BaTiO_3_ composites structured at 6 kV·mm^−1^ with different particle ratios (i.e., 3 vol%, 16 vol% and 29 vol%). The micrographs indicated that the EVA/BaTiO_3_ composites were successfully structured, even at high BaTiO_3_ volume fractions. It was evident, from a structural comparison between the 0–3 and 1–3 composites, that the dielectrophoresis process, as well as the filler content, played a significant role in the resulting structure. Indeed, upon applying an external electric field, the ceramic particles became polarized, resulting in dipole interactions that tended to a form the columnar structure. As the filler volume fraction increased, the fillers lost some of their mobility with the increasing viscosity. This resulted in denser aligned structures where the columns were separated by smaller gaps, and where the inter-particle distance was smaller. This distance could be estimated experimentally through Van den Ende’s theoretical prediction model of the piezoelectric charge coefficient d*_33_*, which is discussed in Section 4.5.

### 2.4. X-ray Diffraction (XRD)

**Figure 6:**
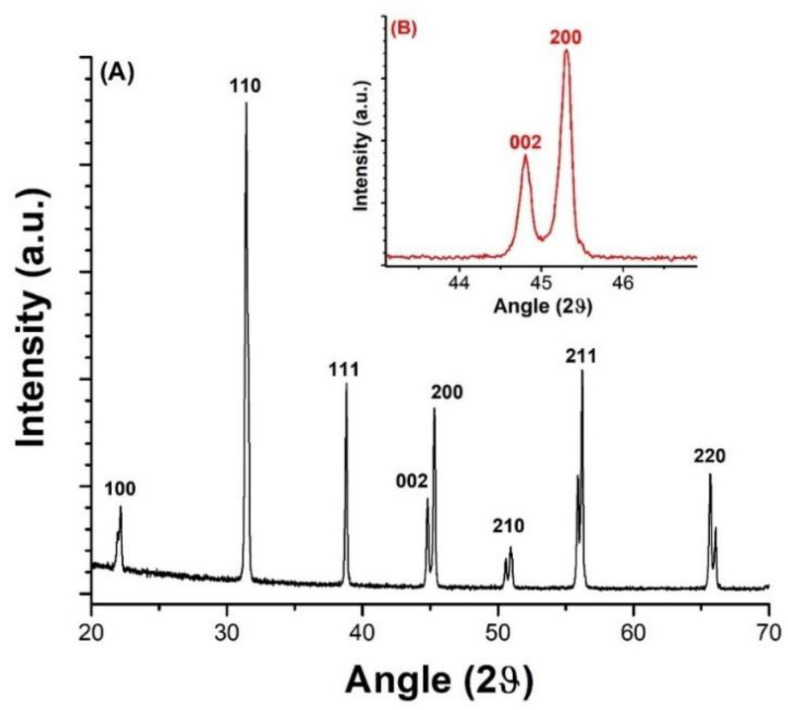
XRD pattern of BaTiO_3_ nanoparticles: (**A**) in the whole range of 20°–70°, (**B**) zoom-in on the 2θ region of 44° and 46°.

The XRD analysis was performed to verify the chemical nature and crystallographic structure of the BaTiO_3_ fillers, as illustrated in Figure 6A. The reported peaks could be indexed primarily as a tetragonal crystal structure [57]. The peaks at 22.2°, 31.4°, 38.8°, 45°, 50.7°, 56°, 65.8° were attributed to (100), (110), (111), (200), (210), (211) and (220) planes, respectively, which was similar to the analyses found in literature [58]. As displayed in Figure 6B, the peak corresponding to 45° was split into two at 2*θ* = 44.8° for (002) and 2*θ* = 45.3° for (200), indicating a tetragonal phase and, consequently, the ferroelectric nature of the BaTiO_3_ nanoparticles [59].

### 2.5. Dynamic Dielectric Spectroscopy

**Figure 7:**
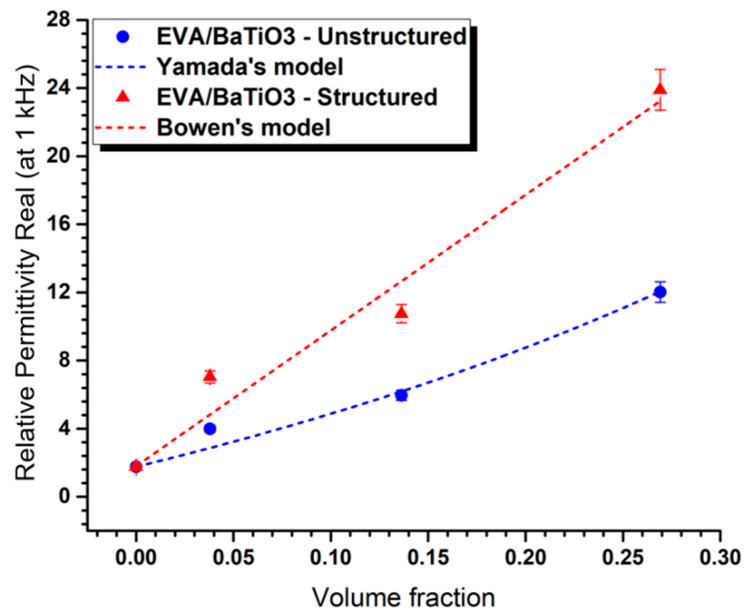
Experimental permittivity values for both structured and unstructured composites compared with Yamada (0–3 connectivity) and Bowen’s (1–3 connectivity) models.

The permittivity of the samples was measured over a frequency range of 1 Hz to 1 MHz, and the effective permittivity of the composites was reported at 1 kHz. The results presented in Table 2 and Figure 7 show the experimental permittivity values of EVA/BaTiO_3_ structured and unstructured composites over different filler contents. In Figure 7, the experimental results of both 0–3 and 1–3 structures were also compared with the theoretical prediction models given by Yamada et al. (for 0–3 connectivity) and Bowen et al., (for 1–3 connectivity) [28,60]. On one hand, Yamada’s theoretical model describes the dielectric permittivity of 0–3 connectivity composites as a function of their filler content. The fillers are considered as ellipsoidal and homogeneously dispersed in the polymer matrix. Yamada’s model is described by the following equation:

(3) $$\varepsilon_{0–3}=\varepsilon_{m}\left(1+\frac{n\varphi \left(\varepsilon_{c}-\varepsilon_{m}\right)}{n\varepsilon_{m}+\left(\varepsilon_{c}-\varepsilon_{m}\right)\left(1-\varphi \right)}\right)$$

where, $\varepsilon_{m}$ and $\varepsilon_{c}$ are the real part of the permittivity of the polymer matrix and the ceramic fillers, respectively; φ is the filler’s volume fraction and n is the parameter attributed to the shape of the ellipsoidal particles. The value of n was obtained by fitting Yamada’s the model to the experimental data (n=14). 

On the other hand, Bowen’s model describes the dielectric permittivity of 1–3 connectivity composites along the alignment direction, where the fillers are represented as cubic shaped and arranged in a columnar structure. Bowen’s model is given by the following equation:

(4) $$\varepsilon_{1–3}=\varphi \left(\frac{r\varepsilon_{c}\varepsilon_{m}}{\varepsilon_{c}+\varepsilon_{m}r}\right)+\left(1-\varphi \right)\varepsilon_{m}$$

where r represents the ratio between the fillers’ median size and the average inter-particle distance within each column. The value of r was obtained by fitting Bowen’s model to the experimental data (r=44).

Regardless of the volume fraction, the dielectric constant of the 1–3 samples was revealed to be higher than that of the 0–3 counterparts. As a matter of fact, this improvement, induced by dielectrophoresis, was a consequence of the columnar rearrangement of the fillers within the matrix which substantially affected the electric field’s distribution and led to an increase in permittivity along the alignment direction. Conversely, the particles in the random configuration were more shielded by the polymer matrix than those in the structured configuration and, therefore, exhibited lower permittivity values. 

The experimental results were also found to be in good agreement with the theoretical predicted values for both structured composites with Bowen’s model and their unstructured counterparts with Yamada’s model. 

### 2.6. Piezoelectric Analysis

**Figure 8:**
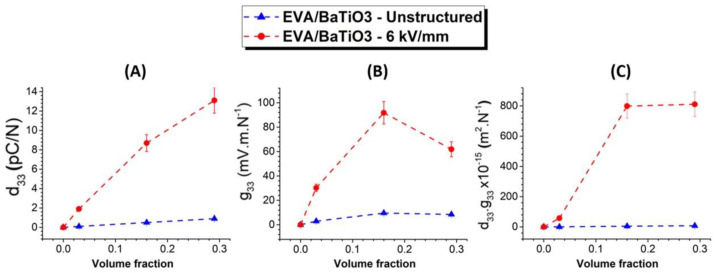
(**A**) Measured piezoelectric charge coefficient (d_33_), (**B**) piezoelectric voltage coefficient, (**C**) transduction coefficient, as a function of the filler content for both structured and unstructured composites.

Table 3 and Figure 8A show the measured piezoelectric charge coefficient (d*_33_*) of structured and unstructured EVA/BaTiO_3_ composites over different filler contents. All characterized samples were poled at 50 °C for 30 min under an electric field of 20 kV·mm^−1^ amplitude.

It can be observed that the d*_33_* values of the structured samples were significantly higher than those of their unstructured counterparts, regardless of the BaTiO_3_ loading level. As mentioned above, the enhancement was related to the rearrangement of the particles into a columnar structure along the field’s direction, resulting in an anisotropic material. In addition, the structural alignment positively affected the polarization process. When applied across the sample’s thickness, the electric field was redistributed non-homogeneously and concentrated in the phase with the lowest dielectric permittivity, due to the boundary conditions at the particle/polymer interface. On one hand, the 1–3 structured composites favored the electric field’s distribution along the alignment direction. This could be explained by the fact that the aligned particles were separated by smaller polymer gaps, allowing for more extensive polarization and, therefore, better activation of the piezoelectric properties. The 0–3 unstructured composites, on the other hand, resulted in a stronger polymeric shielding of the randomly dispersed particles, leading to very weak polarization fields.

Figure 8B depicts the piezoelectric voltage coefficient (g*_33_*) as a function of the filler volume fraction. This coefficient indicates the voltage developed by a piezoelectric material per unit of mechanical stress. While high values of d*_33_* are more relevant in applications involving actuators, high values of g*_33_* are required in materials intended for sensor applications. The *g_33_* coefficient can be determined through the following equation:

(5) $$g_{33}=\frac{d_{33}}{\varepsilon_{0}\varepsilon_{r}}$$

where, $\varepsilon_{0}$ and $\varepsilon_{r}$ are the vacuum permittivity and the samples’ relative permittivity, respectively. 

Figure 8C represents the transduction coefficient (*d_33_*·*g_33_*). This coefficient is proportional to the energy density of a piezoelectric material under an external force. Commonly, this coefficient is used in materials intended for energy harvesting. As expected in Figure 8B,C, both g_33_ and d_33_·g_33_ were revealed to be significantly higher in the structured composites, compared to their unstructured counterparts. regardless of the filler volume fraction. Interestingly, the evolution of d_33_ was quasi-linear, while the g_33_ coefficient encountered a maximum value at 16 vol% (cf. Figure 8A,B). This point represented a filler content threshold, beyond which the sample’s permittivity (i.e., following the percolation law) increased faster than the d_33_. Regarding the d*_33_*·g*_33_* coefficient, at 16 vol%, the structured composites exhibited a 160-fold increase over the unstructured ones, confirming the high benefits of using dielectrophoresis on piezoelectric thermoplastic composites. Above 16 vol%, *d_33_*·*g_33_* tended towards saturation, while g*_33_* declined, giving insight into how to choose an adequate filler volume fraction to optimize the eletromechanical conversion. 

The inter-particle distance within a column can be estimated according to Van den Ende’s model. Equation (6) describes the piezoelectric charge coefficient of quasi-1–3 composites as a function of the filler volume content.

(6) $$d_{33\;structured}=\frac{{\left(1+R\right)}^{2}\times \varepsilon_{m}\times \varphi \times Y_{33\;c}\times d_{33\;c}}{(\varepsilon_{c}+R\varepsilon_{m})\times \left[\left(1+R\varphi \right)\times Y_{33\;c}+\left(1-\varphi \right)\times RY_{m}\right]}$$

where $\varepsilon_{m}$ and $\varepsilon_{c}$ are respectively the relative permittivity of the matrix and the filler; φ is the filler volume content; $Y_{m}$ and $Y_{33\;c}$ indicate the Young’s modulus of the matrix and the filler respectively in the 33 mode [56]. The parameter *R*, reflecting the electric field’s distribution across the composite, indicates the ratio between the particle size and the interparticle distance in the alignment direction.

**Figure 9:**
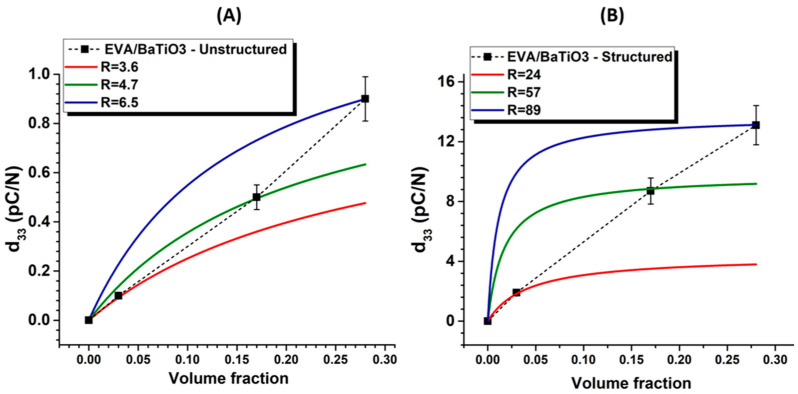
Fitting with Van den Ende’s model for (**A**) EVA/BaTiO_3_ unstructured composites, (**B**) EVA/BaTiO_3_ composites structured at E = 6 kV·mm^−1^.

Van den Ende’s model, initially intended for the 1–3 connectivity, was extended to be used in the 0–3 connectivity, for the purpose of comparison. With each volume fraction, this model was fitted with the experimental data to identify the parameter *R*, from which the overall inter-particle distance within the columns was evaluated (cf. Figure 9A,B). The results showed an increasing *R* with increasing filler volume fraction. The parameter *R* was also revealed to be significantly higher for the structured composites, compared to the unstructured ones. In the model of Van den Ende, *R* is supposed to be constant regardless of the fillers volume faction. Nonetheless, an increase in the filler content reduced the interparticle distance, which, in turn, increased *R*. As a matter of fact, *R* strongly depended on the filler content, which is not the case in Van den Ende’s model. This explains why in Figure 9, the value of *R* was intentionally varied so as to fit the model to the three single-data points. The result clearly showed how the parameter *R* depended on the volume fraction and the structuration of the composite. 

**Figure 10:**
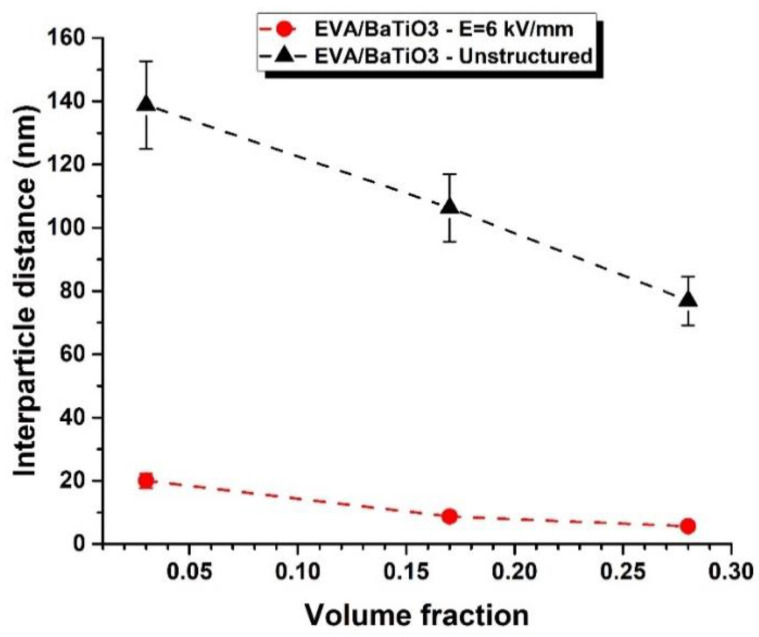
Inter-particle distance in the thickness direction as function of the volume fraction of BaTiO_3_ for structured and unstructured composites, following Van den Ende’s model.

The inter-particle distance, presented in Figure 10, was then calculated and compared for both 0–3 and 1–3 EVA/BaTiO_3_ composites. As expected, the estimated inter-particle distance decreased with increasing content of BaTiO_3_, suggesting that the particles were closer along the sample’s thickness. Figure 10 also revealed an inter-particle distance along the thickness significantly higher for 0–3 composites in comparison with their structured counterparts. Indeed, one of the main consequences of dielectrophoretic structuring is dipolar interactions that tend to bring the filler closer together, thus reducing the overall inter-particle distance. 

**Figure 11:**
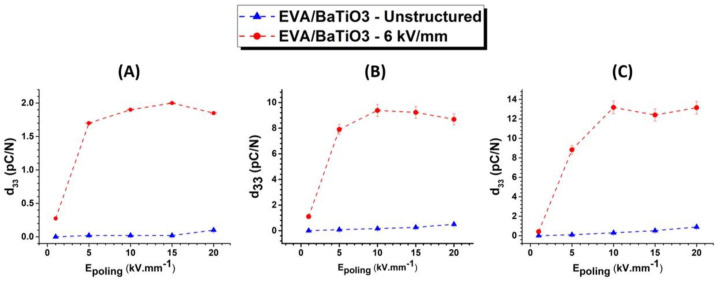
Piezoelectric charge coefficient versus poling electric field of 0–3 EVA/BaTiO_3_ and 1–3 EVA/BaTiO_3_ with a volume fraction of (**A**) 3 vol%, (**B**) 16 vol%, (**C**) 29 vol%.

Finally, in order to identify the saturation of polarization, the dependence of the piezoelectric charge coefficient on the poling electric field was investigated for both structured and unstructured EVA/BaTiO_3_ composites. Each sample was poled for 30 min at 50 °C with a DC electric field of 1, 5, 10, 15 and 20 kV·mm^−1^. The results for the EVA/BaTiO_3_ composites are presented in Figure 11A–C over different volume fractions. As expected, the structured composites exhibited stronger piezoelectric properties compared to the unstructured ones, regardless of which poling level was chosen. It is important to note that the poling field that led to the saturation of the dipole orientation was influenced by the filler connectivity. As previously discussed, in structured composites, the aligned fillers are separated by smaller polymer gaps as a consequence of a shorter inter-particle distance. Consequently, during the poling process, the applied electric field is distributed more efficiently within the composite, allowing for a more extensive polarization. The saturation could, therefore, be achieved at lower electric fields in the structured composites compared to their unstructured counterparts. Thus, the dielectrophoretic structuring resulted in a lower saturation field that ultimately led to higher piezoelectric properties.

### 2.7. Comparison with Existing Piezoelectric Materials

**Figure 12:**
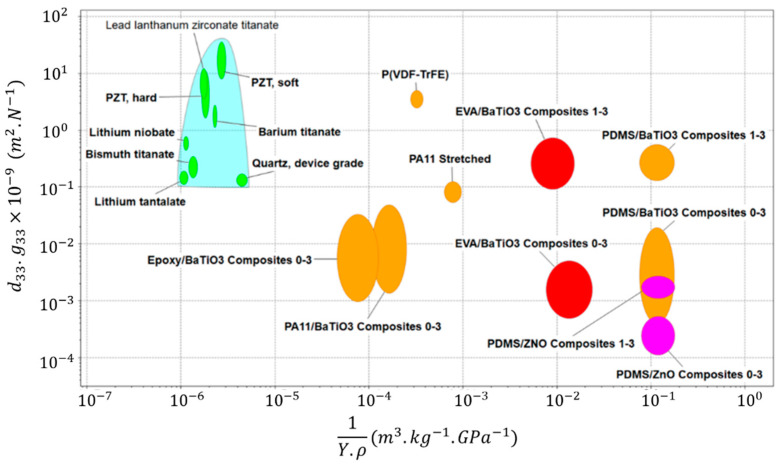
Ashby graph of *d*_33_·*g*_33_ as a function of 1/(*Y*·*ρ*) for various piezoelectric materials.

In order to highlight the inherent potential of the structured thermoplastic piezoelectric composites developed in this study, it was necessary to thoroughly compare them to existing piezoelectric materials found in the literature [10,60,61,62,63]. This comparison was based on selected intrinsic properties, relevant to the development of a high-performance piezoelectric material, such as the piezoelectric voltage/charge coefficients, the density (*ρ*) and Young’s modulus (*Y*). Figure 12 represents an Ashby graph of $d_{33}\cdot g_{33}$ as a function of 1/(*Y·ρ*) for different piezoelectric materials, including bulk ceramic, piezoelectric polymers and piezocomposites. 

As demonstrated in Figure 12, the EVA/BaTiO_3_ structured composites (shown by the red circle) presented a *d*_*33*_·*g*_*33*_ coefficient of the same order of magnitude as some of the denser and stiffer bulk ceramics. The developed composites also competed with other piezoelectric polymers, like P(VDF-TrFE) and PA11, while maintaining a superior weight-elasticity ratio. Particularly, PDMS/BaTiO_3_ elastomer composites, with high flexibility, exhibited similar piezoelectric properties to our developed material. However, they were not thermoplastic and, therefore, could not be recycled after curing. Ultimately, this comparison illustrated the long-term potential behind EVA/BaTiO_3_ composites, in terms of piezoelectric and mechanical properties, while being biocompatible and recyclable thermoplastic materials. 

## 3. Materials and Methods

### 3.1. Material Selection

**Figure 13:**
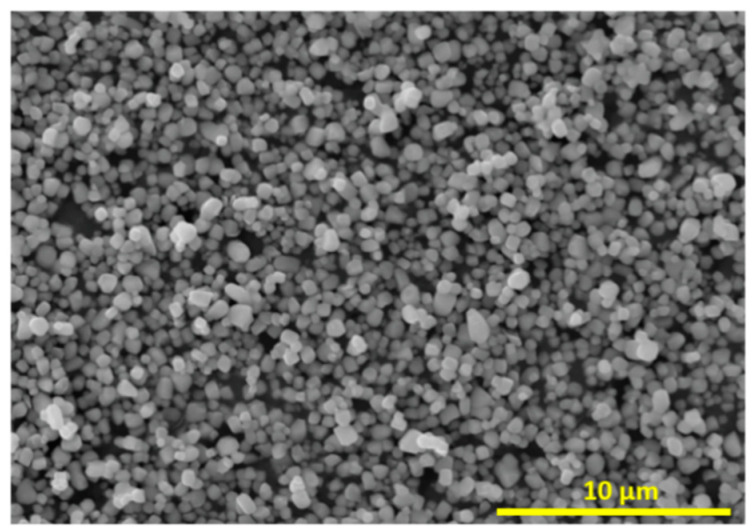
SEM observation of the BaTiO_3_ nanoparticles.

Commercial Barium Titanate (BaTiO_3_) nanoparticles, purchased from US Research Nanomaterials, Inc., were used as piezoelectric fillers. These particles, shown in Figure 13, had a true density of 6.02 g/cm^3^, a specific surface area of 2.06 m^2^/g and a median diameter of 500 nm. Poly(ethylene-co vinyl-acetate) (EVA), purchased from Sigma-Aldrich^®^ (St. Louis, MO, USA), was used as the thermoplastic matrix. This polymer, containing 40 wt% of vinyl acetate, had a density of 0.941 g/cm^3^.

### 3.2. Composite Elaboration

The polymer/ceramic blends were prepared through phase inversion precipitation, described by a ternary phase diagram involving a polymer, a solvent and a non-solvent material. The process initially occurs in the polymer solution obtained by dissolving the polymer in a suitable solvent. As the solution is brought in contact with the non-solvent, the overall composition shifts, resulting in the separation of the solution into a polymer-rich phase and a polymer-poor phase. This behavior corresponds to a critical point where the miscibility gap is crossed, and the polymer begins to precipitate.

Accordingly, the EVA polymer matrix was dissolved in the cyclohexane solvent to obtain a 14 wt% solution. The appropriate amount of BaTiO_3_ particles was then added to the prepared solution to obtain composites with different volume fractions of 3 vol%, 16 vol% and 29 vol%. The solution was then sonicated for 10 min using an ultrasonic probe (Hielscher Ultrasound Technology, UP400S). Subsequently, ethanol was added to the homogeneous solution to induce immiscibility and allow the polymer precipitation. The precipitates were separated from the solvent through vacuum filtration and heated up to 140 °C under a 5-mbar vacuum for 10 min to remove the excess solvent. Subsequently, the BaTiO_3_ content of the composites was determined by TGA.

**Figure 14:**
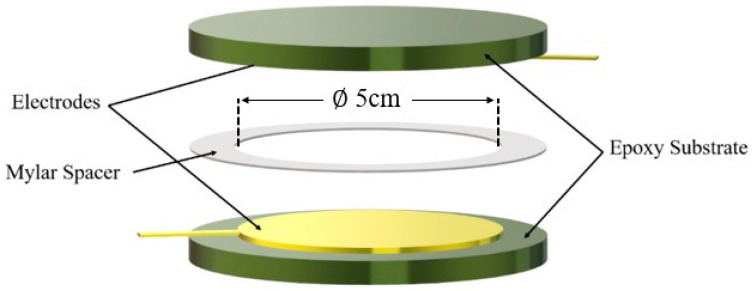
Illustration of the mold used to develop 0–3 and 1–3 composites.

After being cooled down to room temperature, the blend was placed in two identical circular molds of 5 cm diameter, as shown in Figure 14, and heated up again to 140 °C to induce melting. This ensured that the polymer macromolecules acquired enough mobility to allow particle alignment under the influence of the electric field. Upon reaching a stable temperature of 140 °C, the two molds were subjected to a pressure of 1 MPa to ensure a homogeneous sample thickness (~250 µm). The first mold, intended for the unstructured composite, was directly quenched in room-temperature water to fix its structure. The second mold, designed for the structured composite, was connected to a voltage amplifier (TREK 20/20C) coupled to a wave generator. The sample was then subjected to a sinusoidal electric field of 6 kV·mm^−1^ amplitude and 1 Hz frequency for one hour while being maintained under 140 °C at 1 MPa. The applied electric field used in the study (6 kV·mm^−1^) was much lower than the breakdown threshold of the EVA copolymer, which was revealed to equal 77 kV·mm^−1^ approximately [64]. Thus, the electrical breakdown probability of the composites remained small to some extent. Moreover, we implemented a security system to the voltage amplifier that allowed switching off of the circuit once the current exceeded aa limit value that could be defined by the user (around 1–10 mA). Once the aligned structure was successfully formed, both the electric field and pressure were removed, and the sample was quenched in water at room temperature.

### 3.3. Poling Procedure

**Figure 15:**
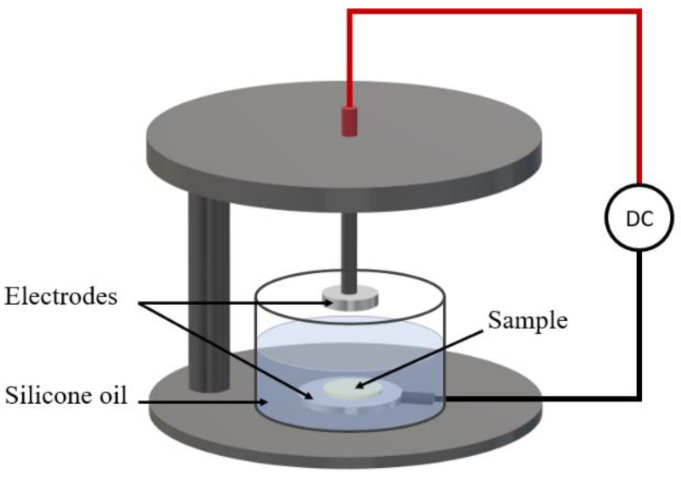
Illustration of the poling setup.

In order to trigger their piezoelectric activity, all prepared samples were poled for 30 min at 50 °C under a high-voltage input. The poling saturation was investigated as a function of the electric field’s amplitude (i.e., from 1 kV·mm^−1^ to 20 kV·mm^−1^). To ensure proper electrical contact, the samples were gold sputtered on both sides using a high-resolution sputter coater (Cressington, 208HR). As shown in Figure 15, the poling process was carried out in a silicon oil bath to avoid the electrical breakdown of air. Subsequently, each sample was placed in a short circuit for 10 min at 50 °C to eliminate the superficial charge carriers accumulated during the process. 

## 4. Characterization Methods

### 4.1. Thermogravimetric Analysis (TGA)

The thermal stability and decomposition of pure EVA and EVA/BaTiO_3_ composites were studied by thermogravimetric analysis (SETARAM 131 EVO) over a temperature range of 25 °C to 800 °C. A 25 mg sample was placed in an aluminum crucible and subjected to the thermal cycle under a nitrogen (N_2_) atmosphere and a heating rate of 10 °C/min. Duplicate runs were also performed to ensure reproducibility.

### 4.2. Differential Scanning Calorimetry (DSC)

**Figure 16:**
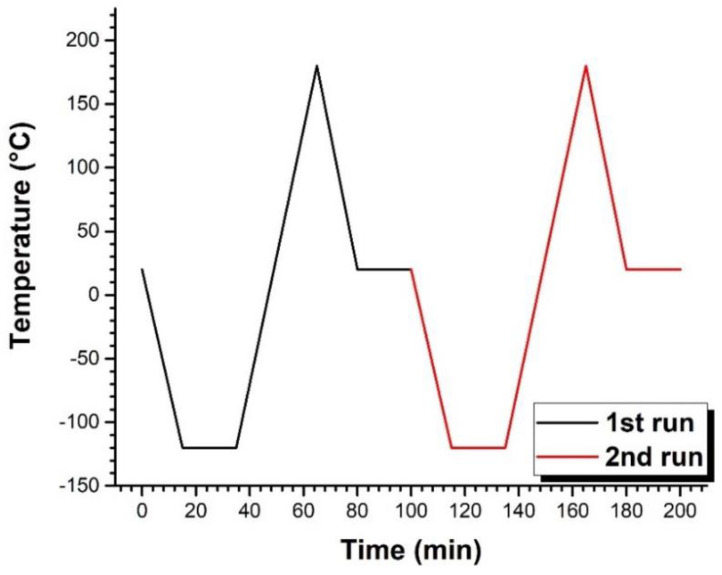
Thermal cycle for DSC measurements of EVA and EVA/BaTiO_3_ composites.

The glass and melting transitions, as well as the crystallinity ratio of pure EVA and EVA/BaTiO_3_ composites, were studied via Differential Scanning Calorimetry (SETERAM DSC131 evo) over a temperature range of −120 °C to 180 °C. The samples were placed in a 100 mL aluminum crucible and subjected to the thermal cycle drawn in Figure 16 under a N_2_ atmosphere. First, the samples were cooled down from room temperature to −120 °C at a cooling rate of 10 °C/min, then stabilized at −120 °C for 20 min. Subsequently, they were heated up to 180 °C and, then, cooled down to room temperature at a constant rate of 10 °C/min. Lastly, the samples were stabilized at room temperature for 20 min before the second run was initiated. All analyzed samples were subjected to two identical thermal cycles with the aim of confirming whether or not the structuring/poling process had an impact on the matrix’s physical structure. Duplicate runs were also performed to ensure reproducibility.

### 4.3. Scanning Electron Microscopy (SEM)

The samples’ cross-sections were characterized through scanning electron microscopy (SEM) observations (HITACHI FlexSEM 1000II). For each volume fraction of BaTiO_3_, both structured and unstructured samples were observed and compared. Given the insulating nature of these composites, the cross-sections were gold sputtered to avoid charge accumulation. Moreover, the gold layer was thin enough (few nanometers) to not impair the analysis, even though Au atoms might be detected by the electron beam. 

### 4.4. X-ray Diffraction (XRD)

BaTiO_3_ particles were characterized by Philips X’Pert MRP diffractometer at ambient temperature to assess the crystalline structure. The measurement was carried out in continuous mode, over a range of 20–70° angles, with a carbon filtered CuKa (1.5406 Å) source.

### 4.5. Dynamic Dielectric Spectroscopy

Dielectric characterization of pure EVA and EVA/BaTiO_3_ composites was carried out through the parallel plate capacitor method using a Solartron 1296 dielectric interface coupled to a Solartron 1255 high frequency response analyzer. The dielectric spectrum was measured at room temperature under an external AC voltage of 1 V amplitude and a frequency ranging from 0.1 Hz to 1 MHz. In this configuration, the AC voltage was applied across the sample thickness (particle alignment direction). For each structural type, the real part of their dielectric permittivity was reported at 1 kHz as a function of the BaTiO_3_ volume content. The measurements were performed before the poling process and duplicated to ensure reproducibility.

### 4.6. Piezoelectric Characterization

The piezoelectric charge coefficient, denoted as d_33_ (pC/N), was measured using a Berlincourt piezoelectric meter (APC YE2730). In order to define the saturation of the polarization for both 0–3 and 1–3 connectivity structures, the piezoelectric properties were assessed as a function of the poling electric field (i.e., 1, 5, 10, 15 and 20 kV·mm^−1^). The measurements were duplicated to ensure reproducibility.

## 5. Conclusions

The proposed research focused on the development of an effective manufacturing process of piezoelectric thermoplastic composites, consisting of lead-free ceramic ferroelectric particles (BaTiO_3_) and a semi-crystalline thermoplastic polymer matrix (EVA). To enhance their piezoelectric properties, the connectivity of the ceramic fillers was increased from a 0–3 to a 1–3 connectivity through dielectrophoretic structuring. Piezoelectric properties of structured (1–3 connectivity) composites were significantly improved in comparison with the unstructured ones (0–3 connectivity). The influence of the process was investigated through dielectric and piezoelectric characterization (*d_33_*), as well as structural microscopic observations (SEM). Indeed, by applying an external electric field, the composites’ structure shifted from randomly dispersed fillers into chain-like arrangements of the piezoelectric particles. This structural change consistently led to higher values of the piezoelectric and dielectric constants, confirming the superiority of the structured 1–3 composites over their unstructured counterparts. The particle rearrangement also led to higher polarization and stronger anisotropic properties along the alignment direction. The results obtained were in good agreement with previous studies regarding thermosetting polymer-based composites, like PDMS/BaTiO_3_ and PU/BaTiO_3_ [12,47]. This work involves the development of an innovative and unprecedented process allowing molten-state dielectrophoretic alignment in a thermoplastic matrix. The thermoplastic nature of the composites offers numerous advantages, such as recyclability, shorter manufacturing time, encapsulation properties, and light weight. Such relevant features, together with biocompatibility, make thermoplastic composites extremely interesting for the design of sustainable medical tools. It is believed that advances in processes and materials (ability to be reused or recycled) could somehow reduce the environmental impact of the health system; particularly in the surgical field, a massive source of greenhouse-gas emissions [65,66].

Accordingly, this research is a stepping-stone towards the use of more advanced structural thermoplastic polymers, such as Polycarbonate (PC) or Polyether-ether-ketone (PEEK) for the manufacture of structured thermoplastic piezocomposites. Moreover, it is possible to expand these composites to new fields of application, such as additive manufacturing and 3D printing.

## Figures and Tables

**Table 1:** Glass transition, melting temperatures and enthalpy of melting as a function of the particle ratios.

Mass Fraction	Volume Fraction	Glass Transition Temperature (°C)	Melting Temperature (°C)	Melting Enthalpy (J/g)
0.00	0.00	−21.5	72.7	25.0
0.18	0.03	−18.2	77.5	27.3
0.54	0.16	−17.6	76.3	23.3
0.72	0.29	−19.0	74.2	24.6

**Table 2:** Relative dielectric permittivity at 1 kHz, as a function of BaTiO_3_ volume content for unstructured 0–3 configuration, and dielectrophoretically structured 1–3 configuration.

Volume Fraction	Relative Permittivity EVA/BaTiO_3_ Unstructured	Relative Permittivity EVA/BaTiO_3_ Structured at E = 6 kV·mm^−1^
0.00	1.8	1.8
0.03	3.9	7.1
0.16	5.9	10.7
0.29	12.0	23.9

**Table 3:** Piezoelectric charge coefficient (*d*_33_) versus filler volume fraction of structured (1–3) and unstructured (0–3) EVA/BaTiO_3_ composites poled at 20 kV·mm^−1^.

Volume Fraction	d_33_ (pC.N^−1^) EVA/BaTiO_3_ Unstructured	d_33_ (pC.N^−1^) EVA/BaTiO_3_ Structured at E = 6 kV·mm^−1^
0.00	0.0	0.0
0.03	0.1	1.9
0.16	0.5	8.7
0.29	0.9	13.1

## Data Availability

Not applicable.
